# The degree of social difficulties experienced by cancer patients and their spouses

**DOI:** 10.1186/s12904-018-0338-9

**Published:** 2018-06-08

**Authors:** Takashi Takeuchi, Kanako Ichikura, Kanako Amano, Wakana Takeshita, Kazuho Hisamura

**Affiliations:** 1 Section of Psychiatry and Behavioral Sciences, Graduate School of Medical and Dental Sciences, Tokyo Medical and Dental University, 1-5-45 Yushima, Bunkyo-ku, Tokyo, 113-8519 Japan; 2 Section of Liaison Psychiatry and Palliative Medicine, Graduate School of Medical and Dental Sciences, Tokyo Medical and Dental University, Tokyo, Japan; 3 Graduate School of Human Sciences, Waseda University, Saitama, Japan; 4 Department of Medical Oncology, Kanazawa Medical University, Ishikawa, Japan; 5 Department of Health Science, School of Allied Health Sciences, Kitasato University, Kanagawa, Japan

**Keywords:** Social problems, Cancer, Patients, Spouses, Difficulties, Internet survey

## Abstract

**Background:**

Although recent studies have increasingly reported physical and psychological problems associated with cancer and its treatment, social problems of cancer patients and their families have not been sufficiently elucidated. The present study aimed to identify cancer-associated social problems from the perspectives of both patients and their spouses and to compare and analyze differences in their problems.

**Methods:**

This was a cross-sectional internet-based study. Subjects were 259 patients who developed cancer within the previous five years and 259 patients’ spouses; the data were derived from two surveys in 2010 (patients) and 2016 (spouses) whose participants were not part of the same dyad but matched by propensity scores, estimated for age, sex, and the presence or absence of recurrence. We investigated the social difficulties of cancer patients and patients’ spouses. Regarding social difficulties experienced by cancer patients and spouses, the 60 patient survey items were categorized into 14 labels by the Jiro Kawakita (KJ) method, which is a qualitative synthesis method developed by Kawakita to classify categorical data.

**Results:**

Although patients had higher scores on most subcategories, young spouses aged 39 or younger and female spouses had difficulty scores as high as the corresponding patients on many subcategories.

**Conclusion:**

Health care providers should show sufficient concern for both patients and their spouses, particularly young and female spouses.

## Background

The diagnosis and treatment of cancer often not only impose physical and mental distress on patients, but also substantially change their daily lives. Consequently, patients often face problems in various aspects of social life, such as family life, relationships with people around them, work, income, leisure activities, and relationships with health care providers [1–5]. Because of current advances in medical technology and availability of outpatient care, the length of hospital stay has been reduced for cancer patients. Because terminal home care is expected to be further promoted in the future, it is assumed that the main site of cancer patient care will increasingly be shifted from hospitals to homes. Under these circumstances, cancer patients and their families are required to deal with various social problems arising as the disease and treatment progress.

Recent studies have increasingly reported on physical problems associated with cancer and its treatment and psychological problems, such as anxiety and depression. As several studies have indicated that social problems experienced by cancer patients have an important impact on their mental health and quality of life [6–10], the need for support for their social problems, as well as the mental care of patients, has been increasingly recognized. However, the reality of the social problems experienced by the families who support cancer patients has not yet been sufficiently understood. Spouse caregivers provide the most extensive and comprehensive care, maintain the caregiver role longer, tolerate greater levels of disability than other caregivers, and experience more severe lifestyle adjustments [11]. The spouse is the primary informal caregiver for cancer patients, and can experience high levels of stress, potential burnout, depressive symptoms, marital distress, poor health, and unmet needs [12, 13].

Thus, the present study aimed to investigate the degree of social difficulties experienced by cancer patients and their spouses, to identify cancer-associated social problems from the perspectives of both patients and their spouses, and to compare and analyze differences in their problems.

## Methods

### Study design and subjects

This is a cross-sectional study using internet-based surveys. The internet survey company we used is a Japanese company specializing in academic research. For the patient survey, in January 2010, we screened all registered members of the panel of internet-survey company A and selected those who developed cancer within the previous 5 years and experienced social problems. For the spouse survey, in the same manner in November 2016, we selected spouses of patients who developed cancer within the previous 5 years. Subjects were 259 patients and 259 patients’ spouses and all cancer types were selected.

### Assessment indicators

#### The degree of social difficulties experienced by cancer patients and spouses

The list of social difficulties experienced by cancer patients and spouses was prepared based on a list of patients’ problems developed from the results of a qualitative survey asking, “What bothers you as a cancer patient?” in our previous study [14]. In this previous study, the list of social difficulties of patients was developed through discussion between an oncology social worker and a psychiatrist who reviewed articles published in Japan and other countries on social problems, distress, stressors, and patients’ unmet needs. This list contains 60 items, on which patients are asked to answer the question, “Have you ever experienced any difficulty concerning the following matters at home, at work, or in your community because of your disease and treatment?” by choosing one of the following 6 options: It has been very difficult; it has been fairly difficult; it has not been very difficult; it has never been difficult; I do not know; and not applicable (Table 1). In the present study, to investigate difficulty perceived by spouses regarding the social problems of patients, we asked spouses to answer the question, “Have you ever experienced any difficulty concerning the following matters at home, at work, or in your community because of the disease (cancer) and treatment of the patient (your spouse)?” on the 60 items in the same manner. To use the 60 items for the spouse survey, the word “you” was replaced with “the patient,” and “your family” with “you.” For both the patient and spouse surveys, the options, “It has never been difficult,” “I do not know,” and “not applicable,” were combined as “It has never been difficult,” and the survey results were statistically analyzed as those of a four-choice survey: “It has never been difficult (0 points),” “It has not been very difficult (1 point),” “It has been fairly difficult (2 points),” and “It has been very difficult (3 points).” Furthermore, in the present study, these 60 items were first classified by the Jiro Kawakita (KJ) method (Affinity Diagram). The KJ method is a qualitative synthesis method developed to classify categorical data by Kawakita [15, 16]. Specifically, two clinical psychologists independently classified the 60 items. Then, items with mismatched labels were labeled through discussion between the psychologists. Next, a group led by a psychiatrist that included clinical psychologists routinely involved in supporting cancer patients reviewed the list of labeled items, made corrections to the arrangement of items and wording of the labels, and finalized the labels. Second, these labels were used as the subcategories of this list. For each subcategory, α coefficient was calculated to evaluate internal consistency. Finally, scores on each subcategory were calculated by dividing the total score by the number of items in each subcategory. This research was not a qualitative study. To adjust the scale of the social problems created by Hisamura et al., we used the KJ method which is a qualitative synthesis method. We chose this approach because we study social systems and social problems peculiar to Japan that cannot be measured by existing social problem scales.

**Table 1:** Classification of difficulties according to the Jiro Kawakita method

Label	Item
1. Difficulty in performing activities of daily living	Outpatient examinations and regular hospital visits
(α = .847)	Going out and transportation methods (including the use of public transportation systems)
	Taking care of myself (e.g., eating, bathing, toileting, and dressing)
	Household chores (e.g., house cleaning, laundry, meal preparation, and grocery shopping)
	Sex life
	Your disease and treatment have made hobbies, pastimes, and social activities less enjoyable.
2. Difficulty in seeking expert advice on the disease state and treatment	Admission to, discharge from, and transfer from a hospital
(α = .909)	Consulting a specialist other than your attending physician about your disease state and treatment
	You and your family cannot receive necessary psychological counseling.
	In case of sudden deterioration of your physical condition, there is no guarantee that you can immediately consult any doctors at the hospital where you are currently treated (or your neighborhood hospitals or clinics).
	Neither you nor your family have any primary care physicians at hospitals or clinics whom you can consult whenever necessary.
	Selection of a hospital (or a physician) that will provide treatment and examinations to you
3. Complaints with health care providers	Health care providers (e.g., physicians and nurses) do not promptly deal with your physical problems.
(α = .932)	Health care providers do not recognize your emotional problems or show any concern.
	Health care providers (e.g., your attending physician, physicians at other departments, your primary care physician, and nurses) do not sufficiently communicate with each other to arrange your treatment and care.
	Before you choose treatment, health care providers do not sufficiently explain the beneficial and adverse effects of each treatment strategy.
	Health care providers do not sufficiently explain the policy or plan of future treatment.
	You cannot talk frankly with your attending physician.
4. Lack of information on treatment and disease state	You cannot obtain enough information on the methods and contents of tests.
(α = .937)	You do not know how to collect information on the treatment of your disease.
	You cannot obtain enough information on various treatment methods.
	You cannot obtain enough information on complementary and alternative medicine (methods that are not regarded as standard treatment at present, such as health food, hot springs, and Qigong).
	You cannot obtain enough information on palliative medicine and care that alleviates pain and distress.
	You cannot obtain enough information on your current disease state and prognosis.
	You cannot obtain enough information on how to treat adverse effects of treatment that you receive and symptoms of your disease.
5. Lack of information on self-care	You cannot obtain enough information on what to keep an eye on in future life.
(α = .909)	You cannot obtain enough information on appropriate nutrition and dietary patterns.
	You cannot obtain enough information on how to deal with anxiety and depression.
6. Conflict over family relationships	Your family do not understand your disease or treatment well and sufficiently cooperate with you.
(α = .898)	Your views on your disease and treatment differ from the views of your family.
	Talking with your family about your disease
	Relationship and communication with your spouse
7. Concerns for family members	Care of your family (e.g., care of your parents, child-rearing, and nursing by your spouse)
(α = .897)	Burden on your family
	You cannot sufficiently take care of anxiety or concerns that your family have.
	Support offered to your family (e.g., help from people around you or public services) is insufficient.
	You cannot sufficiently perform your role at home.
	Your family are overly worried about you.
8. Difficulty in planning life	Planning future life of you and your family
(α = .594)	Developing your plans for marriage, pregnancy, and delivery
9. Conflict over relationships with non-family members	Relationships and communication with your friends and people close to you
(α = .902)	Relationships and communication with your neighbors
	Talking about your disease with people at work or in other social occasions
10. Difficulty in adapting to changes in the social environment	You have been discriminated because of your disease.
(α = .804)	The attitudes of people around you have changed after the onset of your disease.
	Your looks (appearance) have changed.
11. Lack of local support services	You do not have any opportunity to talk with people with similar experiences.
(α = .902)	You are lonely.
	You do not have anyone with whom you can consult or have access to services that offer consultation for the disease and medical care.
12. Difficulty in solving work-related issues	It is difficult to return to and continue work (or study if you are a student).
(α = .892)	It is difficult to take a day off from work (or school if you are a student) for treatment.
	Your disease has adversely affected your promotion at work.
	You have been demoted or transferred to an unimportant position at work.
	You have been asked to retire or fired at work (In case of being self-employed, you have closed your business).
13. Difficulty in making financial arrangements	Medical and living expenses during treatment
(α = .824)	The use of financial services (e.g., loan, medical insurance, and life insurance)
	Management of the properties of yours and your family
14. Lack of information on welfare services available during treatment	You cannot obtain enough information on available welfare services and systems (e.g., nursing-care insurance and welfare services for people with disabilities).
(α = .925)	You cannot obtain enough information on available home-based medical care services (e.g., home-visit medical treatment and nursing).
	You cannot obtain enough information on support for your medical care (e.g., wig, elastic stocking, wheelchair, and special bed).

#### Demographic and clinical variables of the patients

The demographic and clinical data were collected from self-administered surveys of all the study subjects. Specifically, the cancer patients were asked to answer multiple-choice questions on their sex, age, the presence or absence of recurrence, cancer sites, treatment, treatment regimens, state of treatment, academic background, and occupation.

### Analysis methods

First, sex, age group, and presence or absence of recurrence were selected as covariates that should be adjusted based on clinical judgment, and propensity scores for these covariates were estimated using logistic regression models. Based on the calculated propensity scores, the patient and spouse groups were matched in a 1:1 ratio using nearest neighbor matching by sampling without replacement. Frequency distributions of the matched data were generated for background factors in each group to assess the balance among the covariates.

Second, differences in the degree of social difficulties experienced by the patient and spouse group were analyzed by independent two-sample *t*-tests. Furthermore, *t*-tests were also performed to analyze differences in the degree of the difficulties for the following combinations of subgroups: between the younger generation (≤39 years) and middle-aged and older generation (≥40 years), and between male patients/female spouses and female patients/male spouses.

The significance level was set at 5%. SPSS for Windows 23 was used to perform statistical analyses.

## Results

### Subcategories of social difficulties experienced by cancer patients and spouses according to the KJ method (affinity diagram)

According to the KJ method, social difficulties were classified into 14 labels. Specifically, the 60 items were classified into the following subcategories: “difficulty in performing activities of daily living,” “difficulty in seeking expert advice on the disease state and treatment,” “complaints with health care providers,” “lack of information on treatment and disease state,” “lack of information on self-care,” “conflict over family relationships,” “concerns for family members,” “difficulty in planning life,” “conflict over relationships with non-family members,” “difficulty in adapting to changes in the social environment,” “lack of local support services,” “difficulty in solving work-related issues,” “difficulty in making financial arrangements,” and “lack of information on welfare services available during treatment.” The α coefficients for the internal consistency of the subcategories are shown in Table 1.

### Matching for the characteristics and propensity scores of the subjects

**Fig. 1:**
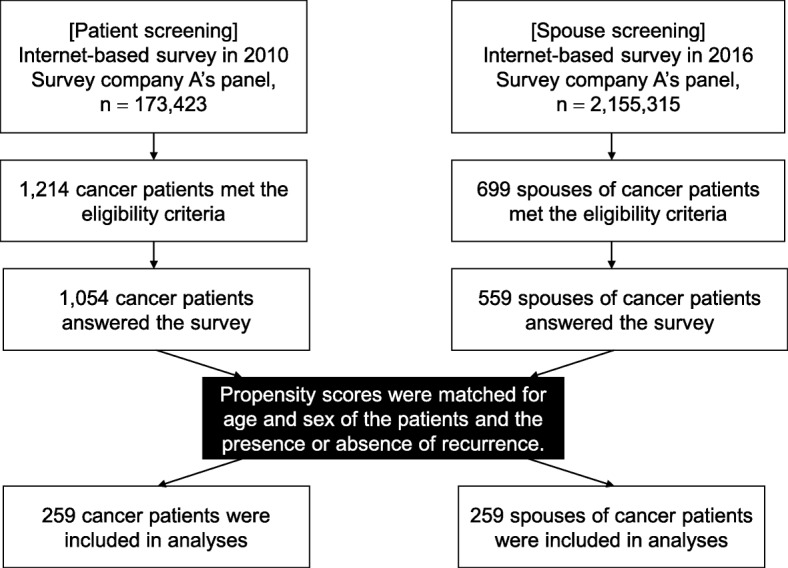
Trial profile

When the patients and spouses were matched based on the propensity scores calculated from logistic regression models, 259 subjects were selected from each of the patient and spouse groups (Fig. 1). Table 2 shows the frequency distributions of the covariates and other background factors for each group. Regarding the covariates, the frequency distributions of sex, age group, and presence or absence of recurrence were completely matched between the patient and spouse groups, confirming that the groups were well balanced and matched. Regarding other background factors, cancer sites varied in both the patient and spouse groups, while most participating patients and patients of most participating spouses were being treated or followed up after the completion of treatment.

**Table 2:** Demographic and clinical data

	Patients *n* (%)	Spouses *n* (%)
Patients’ sex		
Men	128 (49.4)	128 (49.4)
Women	131 (50.6)	131 (50.6)
Patients’ age		
20–29 years	11 (4.2)	11 (4.2)
30–39 years	44 (17.0)	44 (17.0)
40–49 years	67 (25.9)	67 (25.9)
50–59 years	85 (32.8)	85 (32.8)
60- years	52 (20.1)	52 (20.1)
The presence or absence of recurrence		
Primary occurrence	62 (23.9)	62 (23.9)
Recurrence	197 (76.1)	197 (76.1)
Cancer site (including duplicates)		
Lung cancer	34 (13.1)	29 (11.2)
Prostate cancer	25 (9.7)	13 (5.0)
Renal cancer	19 (7.3)	6 (2.3)
Bladder cancer	19 (7.3)	5 (1.9)
Testicular cancer	15 (5.8)	5 (1.9)
Gastric cancer	48 (18.5)	31 (12.0)
Esophageal cancer	20 (7.7)	5 (1.9)
Colorectal cancer	49 (18.9)	50 (19.3)
Liver cancer	17 (6.6)	6 (2.3)
Gallbladder cancer	13 (5.0)	2 (0.8)
Pancreatic cancer	12 (4.6)	3 (1.2)
Breast cancer	79 (30.5)	63 (24.3)
Thyroid cancer	20 (7.7)	6 (2.3)
Head and neck/oral cancer	11 (4.2)	7 (2.7)
Uterine cancer	27 (10.4)	12 (4.6)
Ovarian cancer	18 (6.9)	3 (1.2)
Leukemia	17 (6.6)	8 (3.1)
Malignant lymphoma	18 (6.9)	15 (5.8)
Malignant bone tumor	10 (3.9)	1 (0.4)
Brain tumor	17 (6.6)	1 (0.4)
Skin cancer	14 (5.4)	2 (0.8)
Cancer of unknown primary	5 (1.9)	0 (0.0)
Others	18 (6.9)	24 (9.3)
Treatments (including duplicates)		
Surgery	217 (83.8)	208 (80.3)
Radiation therapy	92 (35.5)	95 (36.7)
Chemotherapy	147 (56.8)	101 (39.0)
Patients’ treatment status		
Patients are currently under cancer treatment	138 (53.3)	118 (45.6)
Cancer was cured, and treatment was completed.	90 (34.7)	120 (46.3)
Cancer is not cured, but aggressive treatment has been completed.	12 (4.6)	12 (4.6)
Others	19 (7.3)	9 (3.5)
Academic background		
Junior high-school graduates	8 (3.1)	10 (3.9)
High-school graduates	68 (26.3)	73 (28.2)
Vocational school/junior college graduates	53 (20.5)	65 (25.1)
Four-year college graduates	130 (50.2)	111 (42.9)
Work		
Full-time employment	98 (37.8)	140 (54.1)
Part-time employment	28 (10.8)	35 (13.5)
Full-time housewife	37 (14.3)	55 (21.2)
Leave of absence because of the disease	15 (5.8)	1 (0.4)
Voluntary retirement because of the disease	21 (8.1)	6 (2.3)
Dismissal (closure of business) because of the disease	11 (4.2)	0 (0.0)
Mandatory retirement	23 (8.9)	12 (4.6)
Unemployed	11 (4.2)	6 (2.3)
Others	15 (5.8)	4 (1.5)

### Differences in the degree of social difficulties experienced by the patient and spouse groups

The *t*-test results showed that the degree of “difficulty in seeking expert advice on the disease state and treatment” was comparable between the patient and spouse groups (*t* = .75, *p* = .45), whereas the degree of difficulty for all the other subcategories was higher in the patient than the spouse group (Table 3).

**Table 3:** Differences in difficulty between the patients and spouses (patients, *n* = 259; spouses, *n* = 259)

	Patients		Spouses			
	M	SD	M	SD	*t*	*p*
1. Difficulty in performing activities of daily living	2.14	.74	1.80	.66	5.48	.00^*^
2. Difficulty in seeking expert advice on the disease state and treatment	1.87	.82	1.82	.68	.75	.45
3. Complaint with health care providers	1.89	.81	1.71	.66	2.67	.01^*^
4. Lack of information on treatment and disease state	1.99	.80	1.76	.70	3.40	.00^*^
5. Lack of information on self-care	2.02	.86	1.77	.76	3.45	.00^*^
6. Conflict over family relationships	1.88	.82	1.72	.70	2.36	.02^*^
7. Concerns for family members	2.04	.80	1.86	.70	2.72	.01^*^
8. Difficulty in planning life	2.04	.85	1.78	.76	3.67	.00^*^
9. Conflict over relationships with non-family members	1.92	.82	1.71	.79	3.03	.00^*^
10. Difficulty in adapting to changes in the social environment	2.05	.80	1.68	.73	5.46	.00^*^
11. Lack of local support services	2.08	.92	1.81	.84	3.47	.00^*^
12. Difficulty in solving work-related issues	1.84	.85	1.67	.73	2.41	.02^*^
13. Difficulty in making financial arrangements	2.16	.90	1.87	.81	3.86	.00^*^
14. Lack of information on welfare services available during treatment	1.80	.91	1.63	.76	2.31	.02^*^

*p < 0.05

Meanwhile, in the younger subgroups (≤39 years), the mean difficulty scores in the spouses increased for all subcategories except “difficulty in adapting to changes in the social environment.” The degree of difficulty was comparable between patients and spouses (Table 4).

**Table 4:** Differences in difficulty between the patients and spouses in the adolescents’ and young adults’ generation (patients, n = 55; spouses, *n* = 55)

	Patients		Spouses			
	M	SD	M	SD	*t*	*p*
1. Difficulty in performing activities of daily living	2.37	.88	2.21	.84	.94	.35
2. Difficulty in seeking expert advice on the disease state and treatment	2.07	1.06	2.18	.82	−.64	.53
3. Complaints with health care providers	2.08	1.02	2.10	.75	−.90	.93
4. Lack of information on treatment and disease state	2.17	1.02	2.10	.84	.41	.68
5. Lack of information on self-care	2.26	1.10	2.08	.90	.92	.36
6. Conflict over family relationships	2.09	.99	2.06	.89	.15	.88
7. Concerns for family members	2.26	.92	2.22	.80	.26	.80
8. Difficulty in planning life	2.57	.96	2.31	1.00	1.41	.16
9. Conflict over relationships with non-family members	2.24	.99	2.10	.95	.79	.43
10. Difficulty in adapting to changes in the social environment	2.42	.95	1.95	.91	2.67	.01^*^
11. Lack of local support services	2.30	1.10	2.19	.94	.53	.60
12. Difficulty in solving work-related issues	2.17	1.03	2.12	.91	.26	.80
13. Difficulty in making financial arrangements	2.44	.93	2.22	.95	1.22	.23
14. Lack of information on welfare services available during treatment	1.99	1.09	1.93	.94	.34	.73

*p < 0.05

In the male patient/female spouse subgroups, the mean difficulty scores in the spouses increased for the following subcategories: “difficulty in seeking expert advice on the disease state and treatment,” “complaints with health care providers,” “lack of information on treatment and disease state,” “lack of information on self-care,” “concerns for family members,” “lack of local support services,” “difficulty in making financial arrangements,” and “lack of information on welfare services available during treatment.” The degree of difficulty for these subcategories was comparable between patients and spouses (Table 5).

**Table 5:** Differences in difficulty between male patients and female spouses (patients, *n* = 128; spouses, *n* = 128)

	Patients		Spouses			
	M	SD	M	SD	*t*	*p*
1. Difficulty in performing activities of daily living	2.15	.78	1.75	.70	4.34	.00^*^
2. Difficulty in seeking expert advice on the disease state and treatment	1.84	.80	1.81	.72	.33	.74
3. Complaints with health care providers	1.86	.81	1.70	.70	1.70	.09
4. Lack of information on treatment and disease state	1.94	.79	1.77	.77	1.81	.07
5. Lack of information on self-care	1.92	.86	1.79	.83	1.26	.21
6. Conflict over family relationships	1.88	.82	1.73	.70	1.60	.11
7. Concerns for family members	2.06	.79	1.88	.74	1.84	.07
8. Difficulty in planning life	2.05	.83	1.76	.74	2.99	.00^*^
9. Conflict over relationships with non-family members	1.91	.80	1.62	.79	2.99	.00^*^
10. Difficulty in adapting to changes in the social environment	2.04	.87	1.66	.79	3.65	.00^*^
11. Lack of local support services	1.98	.88	1.82	.91	1.52	.13
12. Difficulty in solving work-related issues	1.94	.86	1.66	.70	2.90	.00^*^
13. Difficulty in making financial arrangements	2.24	.93	2.01	.92	1.96	.05
14. Lack of information on welfare services available during treatment	1.80	.91	1.66	.82	1.30	.19

*p < 0.05

## Discussion

In the present study, difficulty for cancer patients and their spouses in dealing with social problems was investigated and compared. Because there have been few studies that directly examined these problems, we would like to discuss them in associations with their consequences, such as depression, distress, morbidity, burden, unmet need, and decreased quality of life.

### Comparison of difficulty between patients and spouses

The degree of difficulty was higher in the patient than the spouse group for all subcategories except “difficulty in seeking expert advice on the disease state and treatment (lack of opportunities to consult for patient transfer arrangement, hospital selection, second opinion, psychological counseling, etc.).” Patients and partners are interdependent in that cancer impacts on their shared life, both emotionally and practically. However, no conclusion has been reached on whose distress is more severe because there are conflicting reports. While some reports indicate that distress severity is comparable between patients and their spouses or partners, other reports indicate that patients’ distress is more severe than that of their spouses or partners, and there are even reports indicating that distress of spouses or partners is more severe than that of patients [17]. Hodges et al. [18] investigated mental distress of spouses or partners of patients over the course of disease and confirmed that the distress of spouses or partners significantly correlated with that of patients. Then, they indicated that the mental distress of spouses or partners gradually increases after diagnosis and becomes more strongly correlated with that of patients. Because currently treated patients and spouses of such patients accounted for 50% of the subjects in the present study, the degree of difficulty perceived by the spouses might not have been as high as that of difficulty perceived by the patients. However, we assume that the degree of “difficulty in seeking expert advice on the disease state and treatment” in the spouses was as high as that in the patients because spouses were greatly involved in treatment of patients soon after diagnosis.

### Difficulty perceived by the young spouses

In the young subgroup, the degree of difficulty perceived by the spouses was as high as that of difficulty perceived by the patients for all subcategories except “difficulty in adapting to changes in the social environment (I feel that my appearance has changed or that I am treated differently).” People aged 39 years or younger, who were classified as the younger generation in the present study, are called “adolescents and young adults (AYA).” Cancer patients in the AYA generation experience, after diagnosis and treatment, not only difficulties associated with social relationships, work, academic background, property, etc., but also many physical and psychosocial problems, such as interruptions to romantic and/or intimate relationships, reconsideration of family planning, infertility, and body image dissatisfaction [19, 20]. For this reason, young caregivers in this generation often seem to feel burdened [21, 22] and to perceive a high degree of difficulty.

### Difficulty perceived by female spouses

For the combination of a male patient and a female spouse, the degree of difficulty perceived by the spouses was as high as that of difficulty perceived by the patients for “difficulty in seeking expert advice on the disease state and treatment (lack of opportunities to consult for patient transfer arrangement, hospital selection, second opinion, psychological counseling, etc.),” “complaint with health care providers,” “lack of information on treatment and disease state,” “lack of information on self-care (lack of knowledge on nutritional needs of patients or how to deal with anxiety),” “concerns for family members,” “lack of local support services,” “difficulty in making financial arrangements,” and “lack of information on welfare services available during treatment (lack of knowledge on the nursing-care insurance system or nursing-care facilities and equipment).” Generally, compared to men who take care of their wives with cancer, women who take care of their husbands with cancer have higher mental morbidity (high levels of distress, depression, and anxiety, and a low level of mental health), physical morbidity (low physical health score, decreased physical function, and loss of physical fitness), and social morbidity (low satisfaction in marriage and limited social support) [12, 23, 24]. Ussher et al. [25] attributed this to the fact that women caregivers are positioned as all-encompassing expert careers, expected to be competent at decision-making, a range of physical caring tasks, and provision of emotional support for the person with cancer. The consequences of this positioning are over-responsibility and self-sacrifice, physical costs and overwhelming emotions. Men caregivers positioned caring as a competency task which they had mastered, and which provided them with satisfaction.

### Limitations

The present study has several limitations. First, the results may have been affected by measurement bias because of the use of data from internet-based surveys. Data reliability is limited by the facts that the participants in these surveys determined whether they met the eligibility criteria and that data on diagnosis, treatment regimens, etc., were self-reported. Thus, in future studies, more accurate medical data need to be collected by conducting questionnaire or interview surveys at medical institutions in combination with review of medical records. However, a merit of an internet-based survey is that it guarantees anonymity and allows participants to respond without worrying about health care providers. Second, because of arrangements for this study and funding issues, several years passed between the patient and spouse surveys, and we were unable to collect data from patient-and-spouse pairs. These facts may also have contributed to measurement bias. The degree of social difficulties differed between the patients and spouses in the present study because both groups might have had different underlying problems. Thus, in future studies, patient-and-spouse pairs need to be targeted and surveyed around the same time. However, in this study, comparison was made while the differences in background problems were minimized as much as possible by matching propensity scores for data on sex, age group, and presence or absence of recurrence. Third, an analysis of non-responder or cancer patients who did not experience social difficulties is key to gaining information about a possible sample bias that might impact study results. This was not done in this study. Fourth, an unstandardized scale was used in this study. That is why we prepared the scale using the KJ method and confirmed the reliability with an α coefficient.

## Conclusions

Health care providers should show sufficient concern for both patients and their spouses, particularly young and female spouses. In other words, it was considered important to change the approach method based on age and sex, and to intervene at the time of diagnosis rather than when the cancer was more advanced.

## Abbreviation

AYA: Adolescents and young adults
